# Characterization of a Newly Isolated Marine Fungus *Aspergillus dimorphicus* for Optimized Production of the Anti-Tumor Agent Wentilactones

**DOI:** 10.3390/md13117040

**Published:** 2015-11-19

**Authors:** Rui Xu, Gang-Ming Xu, Xiao-Ming Li, Chun-Shun Li, Bin-Gui Wang

**Affiliations:** 1 Key Laboratory of Experimental Marine Biology, Institute of Oceanology, Chinese Academy of Sciences, Nanhai Road 7, Qingdao 266071, China; E-Mails: xrxurui@yeah.net (R.X.); aericxu@gmail.com (G.-M.X.); lixmqd@aliyun.com (X.-M.L.); lichunshun@qdio.ac.cn (C.-S.L.); 2 College of Earth Science, University of Chinese Academy of Sciences, Yuquan Road 19A, Beijing 100049, China

**Keywords:** *Aspergillus dimorphicus*, anti-tumor agent, wentilactones, optimization

## Abstract

The potential anti-tumor agent wentilactones were produced by a newly isolated marine fungus *Aspergillus dimorphicus*. This fungus was derived from deep-sea sediment and identified by polyphasic approach, combining phenotypic, molecular, and extrolite profiles. However, wentilactone production was detected only under static cultures with very low yields. In order to improve wentilactone production, culture conditions were optimized using the response surface methodology. Under the optimal static fermentation conditions, the experimental values were closely consistent with the prediction model. The yields of wentilactone A and B were increased about 11-fold to 13.4 and 6.5 mg/L, respectively. The result was further verified by fermentation scale-up for wentilactone production. Moreover, some small-molecule elicitors were found to have capacity of stimulating wentilactone production. To our knowledge, this is first report of optimized production of tetranorlabdane diterpenoids by a deep-sea derived marine fungus. The present study might be valuable for efficient production of wentilactones and fundamental investigation of the anti-tumor mechanism of norditerpenoids.

## 1. Introduction

According to the International Agency for Research on Cancer, lung cancer remains the most common cancer, with estimated 1.8 million new cases (12.9% of total) and 1.6 million deaths (19.4%) globally in 2012 [1]. Non-small-cell lung cancer, with a five-year survival rate of only about 15%, accounts for approximately 80%~85% of all lung cancer cases [2]. The most common liver cancer hepatocellular carcinoma accounts for 70%~85% of the total liver cancers [3,4]. It is estimated nearly 782,000 cases and 745,000 deaths in 2012 worldwide, among which 83% were in developing regions and 50% in China alone [1]. Nowadays, chemotherapy remains an effective and powerful option for cancer therapy [5]. For example, the anti-cancer drug Taxol, a taxane diterpenoid which was originally isolated from the Pacific yew tree, was later found in endophytic fungi and some precursor was also overproduced in *E. coli* [6,7]. However, there are still some limitations and side-effects of conventional chemotherapy drugs. Therefore, more novel effective anti-cancer agents are urgently needed to minimize the cancer incidence and severity [2,4,8].

Diterpenoids are a group of compounds with many pharmaceutical and industrial applications [9,10]. The tetranorditerpenoids wentilactones, which showed plant growth inhibition activity, were originally isolated from *Aspergillus wentii* [11,12]. Our group has been working on the isolation, identification, and bioactivity evaluation of marine-derived natural products from algae and endophytic fungi for many years [13,14]. Recently, we obtained several new tetranorlabdane diterpenoids with anti-cancer activities from a marine alga-derived endophytic fungus *A. wentii* [14]. Among these tetranorditerpenoids, we have proved that wentilactone A (WA) could induce apoptosis and G2/M arrest of human lung carcinoma cells [2]. Wentilactone B (WB) could inhibit proliferation and migration of human hepatoma SMMC-7721 cells [15]. These findings provide potential effectiveness and a theoretical basis for the therapeutic use of wentilactones in the treatment of malignancies [8]. However, the yields of wentilactones in *A. wentii* were relatively low under static cultures, which limited further investigations [14]. There are few reports of enhanced production of norditerpenoids from marine fungus so far [9,16].

In order to obtain fungal strains that could produce more wentilactones and other diterpenoids with similar structures, several marine fungi have been isolated and screened. Recently, we isolated and identified an *Aspergillus dimorphicus* from deep-sea sediment, which could produce slightly higher yields of wentilactones with more structural diversity. There was rare report of *A. dimorphicus* isolates from the marine environment, and only a few natural products have been identified [17]. To get the natural products from marine fungi, culture conditions might be optimized trying to simulate its original habitats. As for these anti-cancer wentilactones in *A. dimorphicus*, they could be detected only under static cultures. How these silent natural products are activated by environmental factors might be interesting for further investigation [16].

For the static fermentation of *A. dimorphicus*, we have tested different media, salinity, initial pH, culture time, temperature, and light conditions. According to the results of single-factor experiments, effects of three factors (salinity, initial pH, and culture time) were chosen for further optimization. The wentilactones production was evaluated by employing response surface methodology (RSM) based on Box-Behnken design [18,19]. The generated conditions were applied to wentilactone production and the results were reevaluated. Furthermore, some small-molecule elicitors were found to have capacity of stimulating the wentilactones production, especially with addition of 3% methanol. We hope that these data presented here could provide new methods for wentilactone production and therefore promote the investigation of the anti-tumor mechanism of tetranorditerpenoids.

## 2. Results and Discussion

### 2.1. Identification of Fungal Strain by Polyphasic Approach

**Figure 1:**
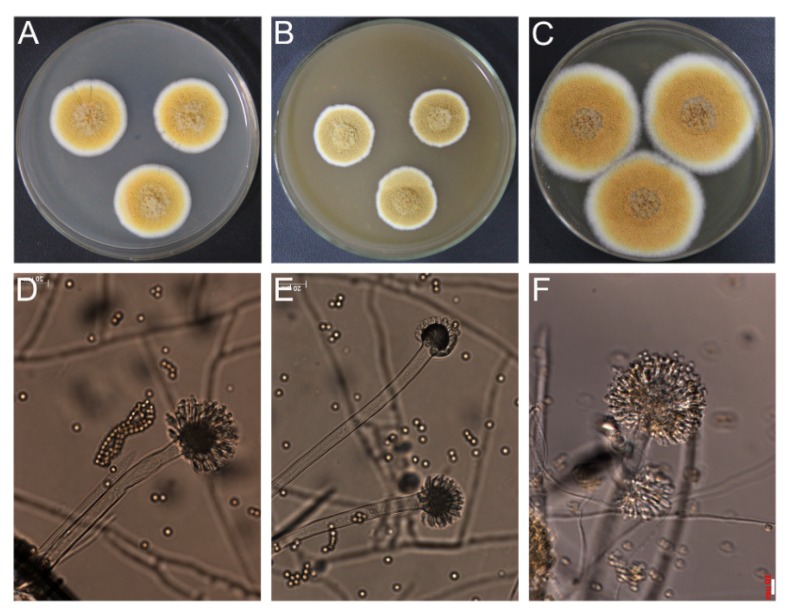
Morphological characteristics of the fungal strain SD317. Colonies on agar media (**A**) CYA; (**B**) MEA; (**C**) YES; (**D**–**F**) conidiophores. Bar, 10 μm.

**Figure 2:**
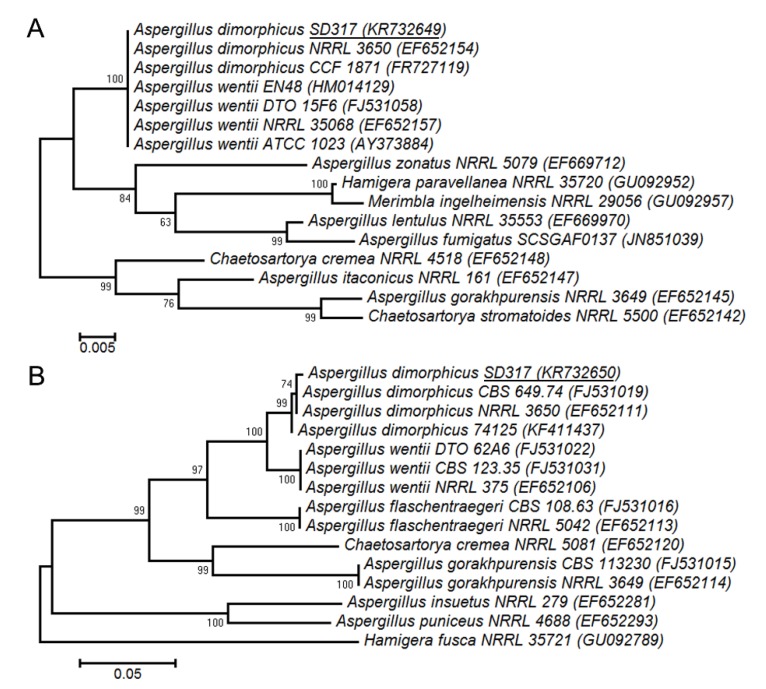
Phylogenetic trees of the fungal strain SD317 and related *Aspergillus* spp. (**A**) Neighbor-joining tree based on the fungal ITS rDNA sequence; (**B**) Neighbor-joining tree based on the beta-tubulin gene sequence. The Kimura two-parameter method was used, with the evolutionary distances bar illustrated and the bootstrap values above the branches.

**Figure 3:**
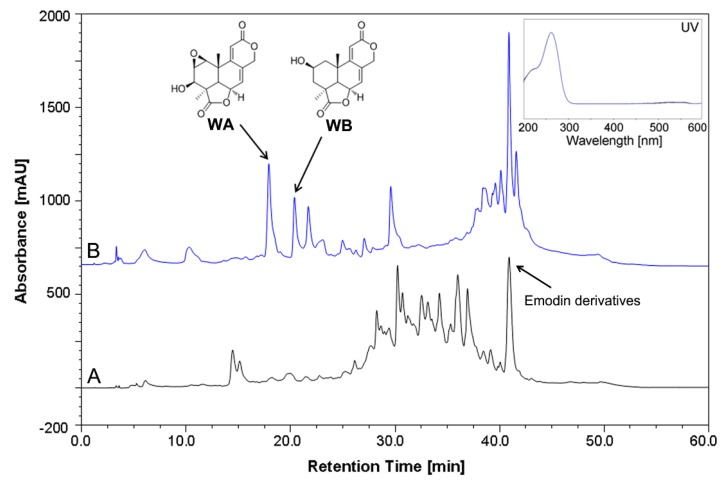
HPLC profiles of the crude extracts of *A. dimorphicus* cultures. (**A**) Extracts under shaking cultures; (**B**) Extracts under static cultures; the structures and peaks of WA and WB are shown with the UV spectrum.

The fungal strain SD317 was recently isolated from deep-sea sediment of the South China Sea. The taxonomic status of this fungus was determined using the polyphasic approach, combining morphological, molecular, and extrolite characteristics. Morphological analysis of the strain indicated that the colonies grown on CYA, MEA, and YES plates have the main characteristics of *Aspergillus* (Figure 1A–F). The colonies reached 2~4 cm diameter in seven days, at first yellowish but later brownish. The mycelia and spores were closely similar to the morphological descriptions of *Aspergillus* section *Cremei*. Genotypic identification of the strain was authenticated by the fungal ITS and beta-tubulin gene partial sequences. After BLAST search in the GenBank database and some of the closely related species selected, the phylogenetic trees were constructed using the MEGA6.0 software [20]. The neighbor-joining tree based on fungal ITS sequence (Figure 2A) showed the clustering of strain SD317 within the *A. dimorphicus* and *A. wentii*, which could not be distinguished separately [21]. However, the phylogenetic tree based on beta-tubulin gene sequence (Figure 2B) showed that strain SD317 was assigned to the *A. dimorphicus*, which is a distinct species [22]. The beta-tubulin gene sequence of strain SD317 has the highest identity value (99%) with several *A. dimorphicus* strains. Strains of *A. wentii* have been reported to produce important metabolites, like aflatoxin, emodin, and wentilactone [11,23]. Extrolite analysis showed that strain SD317 could also produce wentilactones and emodin related derivatives (Figure 3). Based on these combined morphological, molecular, and extrolite porfiles, this marine fungus from deep-sea sediment was identified as *Aspergillus dimorphicus*.

*Aspergillus dimorphicus* was originally reported to be isolated from Indian soils [17]. But after that there were only a few descriptions, some strains even deposited in the GenBank with the name *Aspergillus aff. dimorphicus* [24]. This might due to its difficulty in identification without molecular methods. Some early studies suggested that *A. dimorphicus* might be a synonym of *A. wentii*, which have similar morphological and extrolite characteristics [21]. However, phylogenetic analysis of *Aspergillus* species using DNA sequences from four loci indicated that *A. dimorphicus* was a distinct species [22]. As to genotypic identification of *A. dimorphicus*, it could be easily separated from the closely related species *A. wentii* by more than one gene loci. To our knowledge, this is the first characterization of *A. dimorphicus* from the deep-sea marine environment.

### 2.2. Quantitative Analysis of Wentilactone Production by HPLC

In order to detect these wentilactones’ production in this fungus, an HPLC analysis method was developed for the identification and quantification. The standard calibration curves method was used since we have obtained pure wentilactones [14]. Separations were achieved using conventional C_18_ column with UV detection at 200~400 nm (254 nm for quantification). The mobile phase consists of water and methanol, with methanol varying from 10% to 100% over 40 min (Figure 3). The current method was specific and suitable for routine analysis due to the simplicity, accuracy, and reproducibility. These wentilactones in the crude extracts were determined by this HPLC method for quantitative analysis. There were few reports of HPLC method for norditerpenoid quantification in fungus [11,25]. This method developed in our lab might be useful for quantitative detection of other related tetranorlabdane diterpenoids with similar chromophores.

### 2.3. Effects of Environmental Factors on Wentilactone Production

**Figure 4:**
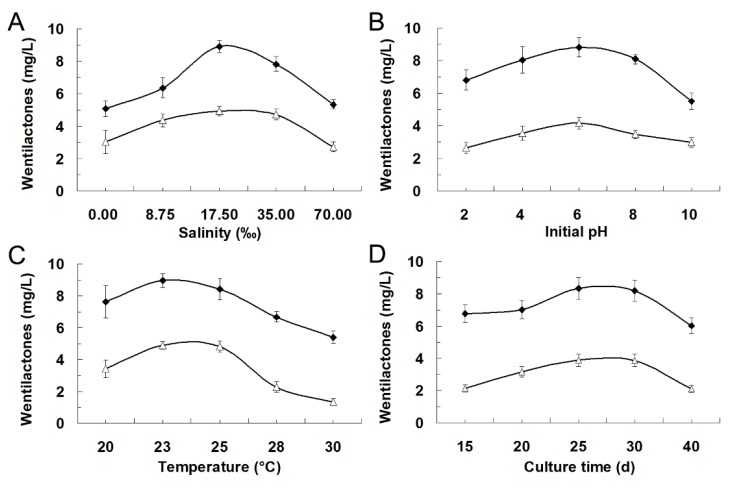
Effects of environmental factors on the wentilactones production. WA (♦); WB (△). (**A**) Salinity; (**B**) initial pH; (**C**) temperature; and (**D**) culture time.

Previously, we found that these wentilactones could only be detected in static cultures (Figure 3). Almost no detectable wentilactones were produced in shaking cultures, which might be silent under these culture conditions. Therefore, different environmental factors were tested to get the optimal culture conditions. During our previous study, this fungus could grow and produce spores well in the modified PDB medium. It could also yield the largest biomass and total extracts. So the modified PDB medium was used for following static fermentation [14]. Then, a series of experiments were carried out with various salinity, initial pH, temperature, and culture time to determine their effects on wentilactone production [26]. As shown in Figure 4A–D, the salinity (17.5‰~35.0‰), initial pH (6~8), temperature (23~25 °C), and culture time (25~30 days), were better for wentilactone production. Salinity might be the main factor that affects the secondary metabolism of marine fungi, which is different from the terrestrial environment [27]. The pH value also affects the biosynthesis and accumulation of fungal products during fermentation [28]. The culture temperature might affect the biosynthesis of wentilactones by fungal growth [17]. Wentilactone production accumulated to high level at later stages, which is consistent with other fungal secondary metabolites.

### 2.4. Optimization of Wentilactone Production by RSM

Based on the results of single-factor experiments, three fermentation factors (initial pH, salinity and culture time) have significant influence on wentilactone production. The effects of initial pH, salinity, and culture time on wentilactone production were further optimized by using RSM. The actual values and the corresponding coded levels of factors and results for each independent variable are given in Table 1. The statistical software Design Expert (Version 8.0.6) was applied to carry out quadratic polynomial regression fitting.

**Table 1:** Box-Behnken design of three factors and three levels and the experimental results.

Runing Order	Variables and Their Coded Values						Yields (mg/L)	
	pH		Salinity (‰)		Cluture Time (d)		Y_a_ (WA)	Y_b_ (WB)
	Code X_1_	X_1_	Code X_2_	X_2_	Code X_3_	X_3_		
1	−1	4.0	−1	0.0	0	25	5.16	1.26
2	1	8.0	1	35.0	0	25	12.28	6.21
3	−1	4.0	1	35.0	0	25	6.28	3.49
4	0	6.0	1	35.0	1	30	10.74	5.64
5	−1	4.0	0	17.5	−1	20	5.66	2.80
6	0	6.0	0	17.5	0	25	12.17	5.77
7	0	6.0	−1	0.0	1	30	5.96	2.97
8	1	8.0	0	17.5	1	30	11.92	6.53
9	0	6.0	−1	0.0	−1	20	3.43	1.50
10	1	8.0	−1	0.0	0	25	7.00	3.16
11	0	6.0	0	17.5	0	25	13.15	6.55
12	0	6.0	0	17.5	0	25	12.93	5.83
13	−1	4.0	0	17.5	1	30	9.09	4.81
14	1	8.0	0	17.5	−1	20	8.14	3.62
15	0	6.0	1	35.0	−1	20	6.44	3.16

#### 2.4.1. Response Surface Analysis of WA

**Figure 5:**
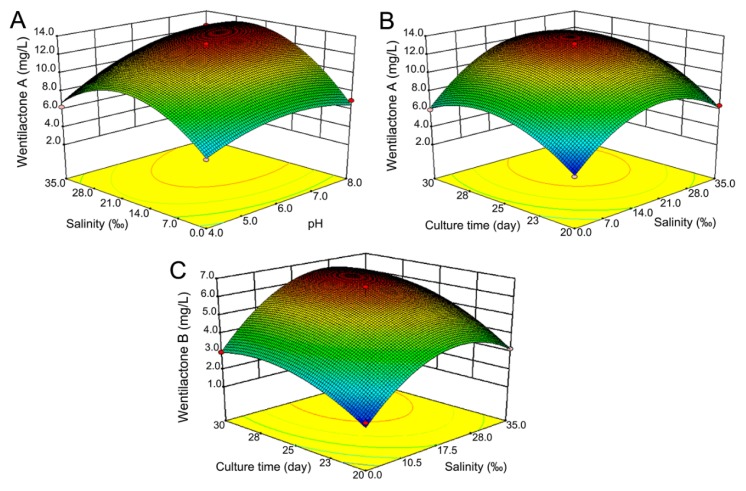
Response surface plots for maximal production of wentilactones. WA: (**A**) pH and salinity; (**B**) salinity and culture time; WB: (**C**) salinity and culture time. The Box-Behnken design of three factors and three levels and experimental results are shown in Table 1.

The regression equation describing the relationship between the WA production and the test variables in coded units is:

*Y*_a_ = 12.75 + 1.64*X*_1_ + 1.77*X*_2_ + 1.75*X*_3_ + 1.04*X*_1_*X*_2_ + 0.44*X*_2_*X*_3_ − 1.50*X*_1_^2^ − 3.56*X*_2_^2^ − 2.54*X*_3_^2^ (1)

where *Y*_a_ is the predicted response of WA production; *X*_1_, *X*_2_, and *X*_3_ are the coded values of pH, salinity, and culture time, respectively. The AVONA analysis results of WA were shown in Table 2. The model *F-*value of 67.53 and *p-*value of 0.0012 indicate that model terms are significant. The lack of fit, with the *F-*value of 1.02, is insignificant. The coefficient of determination (*R*^2^) was calculated as 0.9890 for the WA production, indicating that the statistical model can explain 98.90% of variability in the response (Table 2). Adequate precision measures the signal-to-noise ratio. An adequate precision of 22.764 for the WA production was recorded. The predicted *R*^2^ value is in reasonable agreement with the value of adjusted *R*^2^. This indicates good agreement between the experimental and predicted values for WA production. Thus it could be applied to predict the theoretical WA production. The results of the regression parameter estimate indicated that linear and square terms of pH (*X*_1_), salinity (*X*_2_), and culture time (*X*_3_) values, as well as the interaction term of pH (*X*_1_) and salinity (*X*_2_) were highly significant (*p* *<* 0.05). The relation between factors and WA production was indicated by the 3D surface (Figure 5A,B).

**Table 2:** ANOVA for response surface reduced quadratic model analysis of variance table for WA.

Source	Sum of Squares	Degrees of Freedom	Mean Square	*F*-Value	*p*-Value Prob > *F*
Model	146.79	8	18.35	67.53	<0.0001
*X*_1_	21.64	1	21.64	79.63	0.0001
*X*_2_	25.15	1	25.15	92.57	<0.0001
*X*_3_	24.61	1	24.61	90.58	<0.0001
*X*_1_*X*_2_	4.32	1	4.32	15.90	0.0072
*X*_2_*X*_3_	0.78	1	0.78	2.89	0.1403
*X*_1_^2^	25.93	1	8.36	30.76	0.0015
*X*_2_^2^	86.86	1	46.92	172.69	<0.0001
*X*_3_^2^	23.86	1	23.68	87.82	<0.0001
Residual	1.63	6	0.27		
Lack of fit	1.30	4	0.27	1.02	0.5484
Pure error	0.53	2	0.27		
Cor. total	148.42	14			

#### 2.4.2. Response Surface Analysis of WB

The regression equation describing the relationship between the WB production and the test variables in coded units is:

*Y*_b_ = 6.05 + 0.90*X*_1_ + 1.2*X*_2_ + 1.11*X*_3_ + 0.25*X*_2_*X*_3_ − 0.70*X*_1_^2^ − 1.82*X*_2_^2^ − 0.91*X*_3_^2^ (2)

where *Y*_b_ is the predicted response of WB production; *X*_1_, *X*_2_, and *X*_3_ are the coded values of pH, salinity, and culture time, respectively. The AVONA analysis results for WB were shown in Table 3. The model *F-*value (28.64) and *p-*value (0.0001) indicate that model terms are significant. The lack of fit (*F-*value 1.23) is insignificant. The *R*^2^ was calculated as 0.9663 for the WB production, indicating that the statistical model can explain 96.63% of variability in the response. An adequate precision of 15.248 for WB production was recorded. The results of the regression parameter estimate indicated that linear and square terms of pH (*X*_1_), salinity (*X*_2_), and culture time (*X*_3_) values were highly significant (*p* *<* 0.05), but the interaction terms of the three factors showed less significant effects (*p >* 0.05) on the WB production. The relation between factors and WB production was indicated by the 3D surface (Figure 5C).

By solving the equations, the optimum conditions of cultivation could be initial pH 7.3, salinity 24.5‰, and culture time 27 days. The theoretical highest production of WA and WB could be obtained at 13.9 and 6.9 mg/L. The verification test was done after repeated experiments employing the optimized cultivation conditions. The average actual production of WA and WB reached to 13.4 and 6.5 mg/L, respectively. The results were further verified three times by fermentation scale-up for wentilactone production, each time with 400 flasks totally about 100 L. The purified compounds WA and WB were used for further anti-tumor mechanism studies [2,15]. Therefore, the RSM optimized production of wentilactones was feasible and effective. These might be valuable for fermentation conditions for other norditerpenoid production.

**Table 3:** ANOVA for response surface reduced quadratic model analysis of variance table for WB.

Source	Sum of Squares	Degrees of Freedom	Mean Square	*F*-Value	*p*-Value Prob > *F*
Model	43.54	7	6.22	28.64	0.0001
*X*_1_	6.42	1	6.42	29.57	0.0010
*X*_2_	11.57	1	11.57	53.27	0.0002
*X*_3_	9.87	1	9.87	45.44	0.0003
*X*_2_*X*_3_	0.26	1	0.26	1.19	0.3121
*X*_1_^2^	1.80	1	1.80	8.30	0.0236
*X*_2_^2^	12.26	1	12.26	56.46	0.0001
*X*_3_^2^	3.07	1	3.07	14.11	0.0071
Residual	1.52	7	0.22		
Lack of fit	1.15	5	0.23	1.23	0.5047
Pure error	0.37	2	0.19		
Cor. total	45.06	14			

### 2.5. Elicitation of Wentilactone Production by Small Molecules

**Figure 6:**
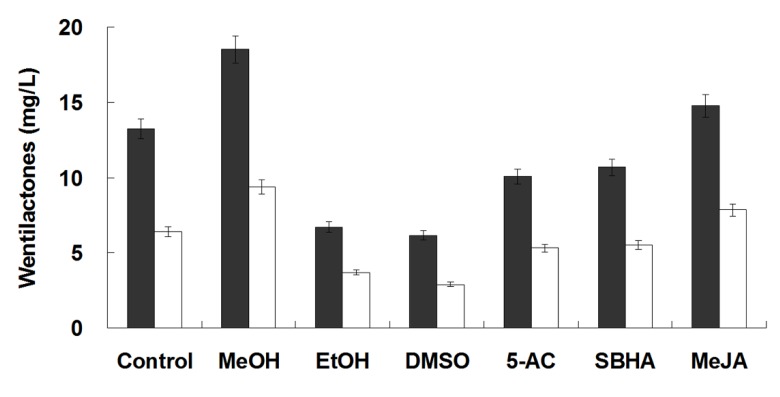
Effects of small-molecule elicitors on the wentilactones production. WA (■); WB (□). Elicitors: MeOH (methanol, 3%), EtOH (ethanol, 3%), DMSO (dimethylsulfoxide, 3%), 5-AC (5-azacytidine, 100 μM), SBHA (suberohydroxamic acid, 100 μM), MeJA (methyl jasmonate, 100 μM).

Three organic solvents (methanol, ethanol, and DMSO) with 3% final concentration, DNA methyltransferase inhibitor 5-AC, histone deacetylase inhibitor SBHA, and plant hormone MeJA with 100 μM were added to the culture for their effects on wentilactone production. With the treatment of 3% methanol on day 15, the production of WA and WB increased to 18.5 and 9.3 mg/L, respectively (Figure 6). Wentilactone yield increased slightly by feeding with MeJA, while a slight decrease was observed with the addition of ethanol, DMSO, 5-AC, and SBHA. Chemical elicitation using small molecules is proving to be an effective way for enhanced production and activation of silent microbial secondary metabolites. Some organic solvents may induce stress response and increases the synthesis of antibiotics [29]. The DNA methyltransferase and histone deacetylase inhibitors might activate unknown cryptic metabolic pathways by epigenetic modifications [16]. Rational design of the optimal time and concentration for the induction of secondary metabolites by addition of small-molecule elicitors still needs detailed investigation. The specific elicitation mechanisms and signal transduction pathways are very interesting for further study.

## 3. Experimental Section

### 3.1. Fungal Strain and Chemicals

The fungus *Aspergillus dimorphicus* SD317 was isolated from deep-sea sediment (South China Sea 2038 m). The fungal ITS (KR732649) and beta-tubulin gene (KR732650) partial sequences of strain SD317 were deposited at the GenBank. The modified Potato Dextrose Broth (PDB) medium was used for liquid static fermentation as described previously [14]. All chemicals used were of analytical grade and commercially available.

### 3.2. Identification of Fungal Strain by Polyphasic Approach

For macro-morphological observations, Czapek Yeast Autolysate (CYA), Malt Extract Autolysate (MEA) agar, and Yeast Extract Sucrose (YES) agar media were used. The isolates were inoculated at three points on each plate of each medium and incubated at 25 °C in the dark for seven days [30]. For micro-morphological observations, microscopic mounts were made in lactic acid from MEA colonies and a drop of alcohol was added to remove air bubbles. Fungal materials were examined using light microscopy (Zeiss Axio Imager 2, Jena, Germany). For genotypic analysis, fungal DNA was extracted using Genomic DNA purification kit (TIANGEN, Beijing, China) according to the instructions. The fungal ITS and beta-tubulin genes were amplified by PCR and sequenced as described previously [30]. After BLAST search of the GenBank database (blast.ncbi.nlm.nih.gov), selected sequences alignment was performed using the CLUSTAL W. Phylogenetic analyses were conducted in MEGA6.0 with the Neighbor-joining method and the Kimura two-parameter model [20]. The bootstrap test was performed with 1000 replications and indicated as percentages. For extrolite analysis, the isolates were grown on CYA and YES at 25 °C for seven days. The extracts were filtered and analyzed by HPLC using UV-VIS detection as described previously [30].

### 3.3. Fungal Fermentation and Metabolite Extraction

Spore suspension was inoculated on Potato Dextrose Agar (PDA) plate and cultured at 28 °C for seven days. The fresh mycelia were inoculated into 1 L flasks containing modified PDB liquid medium. Then the incubated flasks were cultured under different conditions for wentilactone production. After static fermentation, the mycelia and culture broth of were separated and exhaustively extracted with acetone and ethyl acetate, respectively. The combined extracts were subjected to ultrasonic disruption. The metabolites extraction procedure was carried out as described previously [14].

### 3.4. Quantification of Wentilactone Production by HPLC Analysis

The fungal metabolites were analyzed by HPLC (Dionex, Sunnyvale, USA) with C_18_ column (4.6 × 250 mm, 5 μm) and UV-DAD detector (200–600 nm) at 254 nm. A mixture of water and methanol (0~5 min 10% methanol, 5~40 min 10%~100% methanol, 40~50 min 100% methanol) was the mobile phase at a flow rate of 1.0 mL/min. For wentilactone quantification, purified samples (1.0 mg) were dissolved in 1.0 mL of methanol as standard stock solutions, diluted, and analyzed by HPLC using standard calibration curves [14].

### 3.5. Effects of Environmental Factors on Wentilactone Production

The effects of various environmental factors on wentilactone production were studied to determine the optimal culture conditions [26]. A series of experiments were carried out with different salinity (0‰, 8.75‰, 17.5‰, 35.0‰, and 70.0‰), initial pH (2, 4, 6, 8, and 10), temperature (20, 23, 25, 28, and 30 °C), and culture time (15, 20, 25, 30, and 40 days). The extraction procedure, metabolite analyses, and production of wentilactones were performed and determined using the methods described above [14]. All the experiments were performed in triplicate, and the mean values ± standard deviations (SD) were used.

### 3.6. Optimization of Wentilactone Production by RSM

The RSM and Box-Behnken design were employed in further fermentation optimization [19]. According to the results of single-factor experiments, three fermentation factors (initial pH, salinity, and culture time) have significant influence on wentilactone production. Considering the feasible conditions for large scale static fermentation in the lab, a three factor and three level experiment was designed. The actual values and the corresponding coded levels of factors and results for each independent variable are given in Table 1. The experiment consisted of 15 trials including three center points, and the value of dependent response was the mean of three independent experiments. The response variable obtained using RSM was fitted to the second order polynomial equation:

*Y_i_ =* β_0_*+ ∑*β*_i_x_i_ + ∑*β*_ii_x_i_*^2^*+ ∑*β*_ij_x_i_x_j_* (3)

where *Y_i_* is the predicted response; *x_i_* and *x_j_* are input variables which affect the response variable *Y*; β_0_ is the offset term; β_i_ is the *i*th linear coefficient; β_ii_ is the *i*th quadratic coefficient; β_ij_ is the *ij*th interaction coefficient. The second order polynomial coefficients were calculated and analyzed using the statistical software package Design Expert (Version 8.0.6, Stat-Ease Inc., Minneapolis, MN, USA). Statistical analysis of the model was performed by the analysis of variance (ANOVA). All the experiments were performed in triplicates, and the mean values ± SD were used.

### 3.7. Effects of Small-Molecule Elicitors on Wentilactone Production

The effects of different small-molecule elicitors on wentilactone production were also detected during static fermentation under optimized culture conditions [26,29]. Various small-molecule elicitors, including methanol (MeOH 3%), ethanol (EtOH 3%), dimethylsulfoxide (DMSO, 3%), 5-azacytidine (5-AC, 100 μM), suberohydroxamic acid (SBHA, 100 μM), methyl jasmonate (MeJA, 100 μM), were introduced on day 0 or 15 to stimulate wentilactone production. The extraction and metabolite analyses were performed using the methods described above, and wentilactone production was determined by HPLC for quantification.

## 4. Conclusions

In summary, the newly isolated marine fungus *Aspergillus dimorphicus* was identified using a polyphasic approach, combining phenotypic, molecular, and extrolite profiles. Production of the potential anti-tumor agent wentilactones was determined by HPLC quantitatively. The optimal static fermentation conditions were: modified PDB 250 mL/1 L flasks, initial pH 7.3, salinity 24.5‰, culture time 27 days, temperature 23 °C, with the production of WA and WB increased to 13.4 and 6.5 mg/L, respectively. The result was also verified by fermentation scale-up for wentilactone production at room temperature (23 ± 1 °C) and natural light conditions. Furthermore, some small-molecule elicitors could also stimulate wentilactone production, especially with the addition of 3% methanol. To the best of our knowledge, this is the first report of optimized production of tetranorditerpenoids by a deep-sea derived marine fungus. These are valuable for druggability assessment and anti-tumor mechanism studies of these tetranorditerpenoids. Further research would be performed by directed mutations and metabolic engineering to investigate the molecular regulation mechanism of how silent diterpenoids biosynthesis might be activated.
